# Seroprevalence of *Borrelia*, *Anaplasma*, *Bartonella*, *Toxoplasma*, *Mycoplasma*, *Yersinia*, and *Chlamydia* in Human Population from Eastern Poland

**DOI:** 10.3390/pathogens14010096

**Published:** 2025-01-18

**Authors:** Angelina Wójcik-Fatla, Anna Sawczyn-Domańska, Anna Kloc, Joanna Krzowska-Firych, Jacek Sroka

**Affiliations:** 1 Department of Health Biohazards and Parasitology, Institute of Rural Health, Jaczewskiego 2, 20-090 Lublin, Poland; afatla@poczta.onet.pl (A.W.-F.); kloc.anna@imw.lublin.pl (A.K.); jacek.sroka@piwet.pulawy.pl (J.S.); 2 Infectious Diseases Clinic, Institute of Rural Health, Jaczewskiego 2, 20-090 Lublin, Poland; krzowska-firych.joanna@imw.lublin.pl; 3 Department of Parasitology and Invasive Diseases, National Veterinary Research Institute, Aleja Partyzantów 57, 24-100 Puławy, Poland

**Keywords:** seroepidemiologic studies, zoonoses, *Borrelia burgdorferi* sensu lato, *Toxoplasma gondii*, *Anaplasma phagocytophilum*, *Bartonella henselae*, *Yersinia* spp., *Mycoplasma pneumoniae*, *Chlamydia pneumoniae*, Poland

## Abstract

The epidemiological situation related to infectious diseases is influenced by many factors. To monitor actual trends in selected zoonoses, a total of 473 serum samples from farmers, forestry workers, and veterinarians were collected for serological examination. Anti-*Borrelia burgdorferi* sensu lato (s.l.) antibodies were tested with ELISA and Western blot (WB) tests; the detection of anti-*Toxoplasma gondii* antibodies was performed using an enzyme linked fluorescence assay (ELFA). Antibodies to bartonellosis, anaplasmosis, and chlamydiosis were determined by indirect immunofluorescent test (IFA), whereas antibodies to yersiniosis and mycoplasmosis were confirmed in the ELISA test. Positive or borderline results of antibodies against *B. burgdorferi* s.l. in the ELISA test were detected in 33.8% of the study population. The borderline or positive ELISA test results for at least one antibody class were confirmed by WB in 58.7% of cases. The IgG antibodies against *Anaplasma phagocytophilum*, *Toxoplasma gondii*, and *Mycoplasma pneumoniae* were detected in 9.6%, 51.7%, and 63.6% of samples, respectively. Antibodies against *Yersinia* spp., *Bartonella henselae*, and *Chlamydia pneumoniae* were found to vary between 43 and 47%.

## 1. Introduction

Infectious diseases are caused by pathogens transmitted directly from person to person or indirectly through vectors or the environment. In recent decades, better access to health care and modern therapeutics, improved sanitary conditions, and the development of vaccines have led to a significant decline in mortality caused by infectious diseases. Nevertheless, climate change, urbanization, and changes in demographics and land use may also influence the risk of infection, especially with re-emerging diseases [1]. 

Vector-borne diseases are a significant group of infectious diseases. In the Northern Hemisphere, Lyme borreliosis (LB) is the most prevalent tick-borne disease, caused by spirochaetes belonging to the *Borrelia burgdorferi* sensu lato (s.l.) complex. The disease can affect multiple organs, such as the heart, nervous system, joints, or skin [2]. Human granulocytic anaplasmosis (HGA) is an infection caused by *Anaplasma phagocytophilum*, an obligate intracellular gram-negative bacteria transmitted primarily by *Ixodes* ticks. In Europe, the course of the disease is generally mild [3]. *Babesia* infections caused by intraerythrocytic parasites (*B. microti*, *B. divergens*, *B. venatorum*) resemble malaria and is particularly severe in older patients and people with immunodeficiencies [4]. 

*Bartonella* spp. are intracellular bacteria affecting humans and animals that are typically transmitted by insect vectors, mainly fleas. Most human cases are caused by *B. henselae*, *B. quintana*, and *B. bacilliformis*, responsible for Carrion’s disease, cat-scratch disease (CSD), trench fever, bacillary angiomatosis, and peliosis hepatis [5].

*Toxoplasma gondii* is a widespread intracellular protozoan parasite that infects humans and warm-blooded animals. Acute infection is sometimes missed due to mild or unnoticeable clinical symptoms and signs. Primary protozoan infection during pregnancy can lead to congenital infection [6].

According to the European Food Safety Authority (EFSA), yersiniosis is one of the most commonly reported foodborne zoonosis in the European Union [7], caused by *Yersinia enterocolitica* and, less frequently, *Y. pseudotuberculosis*. The disease is most often self-limiting gastroenteritis, although the infection can cause mesenteric lymphadenitis, reactive arthritis, and sepsis [8]. 

Transmission of *Mycoplasma pneumoniae* and *Chlamydia pneumoniae* bacteria occurs mainly through the respiratory tract via aerosol. Both bacteria are associated with various respiratory diseases as well as extra-respiratory manifestations [9,10].

The aim of this study was to determine the seroprevalence of selected infectious diseases in healthy (asymptomatic) residents and workers in rural areas of eastern Poland.

## 2. Materials and Methods

### 2.1. Study Group

The study was conducted in 2019–2020 at the Medical Diagnostic Laboratory of the Department of Health Biohazards and Parasitology at the Institute of Rural Health in Lublin, eastern Poland. The research was carried out as part of a National Health Programme for 2016-2020 financed by the Minister of Health (Poland). The selection of the study group was determined by the guidelines of the Ministry of Health, which included the strategic assumptions of the programme: extending healthy life, improving health and the related quality of life of the population, and reducing social inequalities in health. The programme was addressed to all adult participants (≥18 years) associated with work, employment, or residence in rural, forest, and green areas in cities and suburban areas (including farmers, foresters, and veterinarians). Participation in the research was voluntary after completing and signing the project participant’s declaration (the consent form).

**Figure 1:**
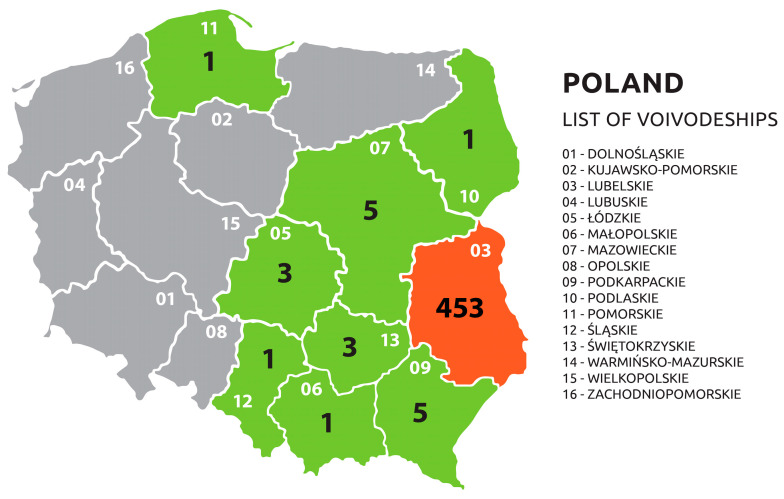
Voivodeships from which the programme participants came, and the number of participants.

The study group consisted of 262 women and 211 men who signed informed consent and responded to a paper survey regarding demographic data (age, gender, and occupation). A total of 473 serum samples were collected over 2 years, 257 samples in 2019 and 216 in 2020, from farmers and rural inhabitants (392), forestry workers (54), and veterinarians (27) living mostly in the eastern regions of Poland. The vast majority (96%) of participants came from Lubelskie Voivodeship (453), with a few people coming from other voivodeships: Podkarpackie (five), Mazowieckie (five), Łódzkie (three) Świętokrzyskie (three), Podlaskie (one), Małopolskie (one), Śląskie (one), and Pomorskie (one) (Figure 1). In 2019, samples were collected from January to December, with the majority collected in July–August (78.6%). In 2020, 95.8% of samples were collected in May. 

### 2.2. Ethics Statement

Ethical approval was obtained from the Ethics Committee of the Institute of Rural Health in Lublin (Approval No. 17/2019) as a part of the National Health Programme for 2016–2020, financed by the Polish Minister of Health in Warsaw (Agreement No.: 6/4/6/NPZ/FRPH/2018/793/209). Each participant was informed orally by a Laboratory employee about the purpose and benefits of participating in the programme, as well as the possible risks associated with blood collection. The participants then signed an informed consent to participate in the programme. The processing of personal data in the laboratory in order to ensure confidentiality was carried out in accordance with the provisions of European law (Regulation (EU) 2016/679 of the European Parliament and of the Council of 27 April 2016 on the protection of natural persons with regard to the processing of personal data and on the free movement of such data, and repealing Directive 95/46/EC (General Data Protection Regulation) (Text with EEA relevance)). Each participant read and signed the Information Clause on the Processing of Personal Data.

### 2.3. Laboratory Analysis

The material for this study was venous blood, from which sera were obtained as diagnostic material. Blood samples were collected in the treatment room at the Specialist Clinic of the Institute of Rural Health in Lublin, Poland, by a qualified nurse under the applicable procedures. Blood from the vein was drawn using a closed system into 9 ml Sarstedt S-Monovette® serum tubes (type of preparation—clotting activator; SARSTEDT, Nümbrecht, Germany) with the vacuum technique. Each tube was labelled with the patient’s name, surname, and personal identification number. Blood samples were transported to the laboratory in the same building and left at room temperature to form a clot (approx. 20 min). After centrifugation for 10 min at 2000× *g* (Sigma Laborzentrifugen GmbH, Osterode am Harz, Germany) at room temperature, the serum was carefully transferred into Eppendorf tubes marked with a bar code and register. The serum was stored until testing at 5 °C (± 3 °C).

All serologic tests were performed by qualified laboratory diagnosticians at the Medical Diagnostic Laboratory of the Department of Health Biohazards and Parasitology at the Institute of Rural Health in Lublin, Poland. Sera were examined for the presence of anti-*B. burgdorferi* s.l. antibodies with the use of Borrelia IgM ELISA (enzyme-linked immunosorbent assay) and Borrelia IgG ELISA (Biomedica, Wien, Austria) tests and immunoblot tests (recomLine Borrelia IgM and recomLine Borrelia IgG, Mikrogen, Neuried, Germany). For ELISA, the sensitivity and specificity of the kits were 96.4 and 100%, respectively. When it comes to Western blot (WB), the sensitivity in patients with Lyme arthritis, acrodermatitis chronica atrophicans, and neuroborreliosis was 96.4%, 100%, and 97.1% for IgM and/or IgG, respectively. The specificity was 100% for the IgM and 99.4% for the IgG tests. 

The detection of anti-*T. gondii* IgM, IgG antibodies, and IgG avidity in serum samples was performed using an enzyme linked fluorescence assay (ELFA) (VIDAS TOXO IgM, VIDAS TOXO IgG II, VIDAS TOXO IgG AVIDITY, BioMérieux, Marcy l’Etoile, France). According to the manufacturer, a high avidity strongly suggests a primary infection lasting longer than 4 months, while low avidity suggests a recent infection. The sensitivity and specificity were 99.5 and 93% for IgM, and 98.35 and 99.77% for IgG, respectively. 

Specific antibodies against *A. phagocytophilum*, *Bartonella* (*B. henselae*, *B. quintana*), and Chlamydia (*C. pneumoniae*, *C. trachomatis*, *C. psittaci*) were determined by indirect immunofluorescent assay (IFA), using the *Bartonella* IFA IgG, Anaplasma phagocytophilum IFA IgG, and Chlamydia MIF IgG assays, respectively (Focus Diagnostics, Cypress, CA, USA). The sensitivity and specificity of the kits were 99.5 and 97.6% for the *Bartonella* kit, 100 and 95.6% for the *Anaplasma* kit, and 99.76 and 96% for the *Chlamydia* kit, respectively.

The recomWell Yersinia IgG ELISA Kit (Mikrogen Diagnostik, Neuried, Germany) with recombinant YOPs (Yersinia outer membrane proteins) antigens for the detection of IgG against *Y. enterocolitica* and *Y. pseudotuberculosis* was used to detect IgG in yersiniosis. The sensitivity and specificity of the kit were 100 and 65%, respectively. IgM and IgG antibodies against M. pneumoniae were detected using ELISA tests (NovaLisa Mycoplasma pneumoniae IgM, IgG, NovaTec Immundiagnostica GmbH, Dietzenbach, Germany). The sensitivity and specificity were 100 and 99.29% for IgM, and >95 and >95% for IgG, respectively. All diagnostic tests were selected based on the recommendations of the Polish Society of Epidemiologists and Infectious Disease Physicians in the field of Lyme disease and toxoplasmosis [11,12], recommendations regarding the selection of the type of serological testing technique (IFA for anaplasmosis) [13], or the availability of tests with IVD for other diseases tested. 

### 2.4. Statistical Analysis

To test associations between year of sampling and prevalence of specific antibodies, we used a 2 × 2 contingency table chi-squared test or Fisher exact test, when needed. The statistical analyses were performed with TIBCO Software Inc. (Palo Alto, CA, USA; 2017) Statistica (data analysis software system, version 13). A *p*-value < 0.05 was considered statistically significant.

## 3. Results

Of the 464 serum samples tested with ELISA, positive or borderline results of antibodies against *B. burgdorferi* s.l. were found in 33.8% (157/464) of the study population (22 participants were tested only by ELISA). Of the 450 serum samples tested with Western blot (WB), 22% (99/450) showed positive results, including eight persons tested only by WB test (Table 1).

For 442 participants, the ELISA and WB tests were performed simultaneously. Among this group, borderline or positive ELISA results for at least one antibody class (*n* = 141) were confirmed by WB for 83 samples, with a seroprevalence rate of 58.7% (83/141; CI: 50.3–67.1%). Of the 90 positive or borderline results in the IgM-ELISA, 43 (47.8%; CI: 36.7–58.0%) were confirmed in the WB, whereas, of the 81 IgG-ELISA borderline or positive results, 54 were confirmed (66.7%; CI: 55.3–76.8%).

Overall, the percentage of seropositive results in the studied population was 18.8% (83/442; CI: 15.2–22.7), confirmed by immunoblot test. In 2019, the immunoblot confirmed the presence of specific anti-*B. burgdorferi* s.l. antibodies in 23.3% (53/227; CI: 18.0–29.4%) serum samples, whereas, in 2020, 14% were confirmed (30/215; CI: 9.6–19.3%). We found a statistically significant difference (*p* < 0.05) in prevalence between 2019 and 2020.

In the current study, among 422 samples tested for the presence of anti-*T. gondii*-specific antibodies, 218 (51.7%) were positive in the IgG class, and an increase in seroprevalence with age was observed. The specific IgM antibodies were detected in six (1.4%) people. Of the 187 sera tested for IgG avidity using the ELFA method, most of the sera (178; 95.2%) were characterized by high IgG avidity, and only one serum (0.53%) had low avidity (Table 2).

*Anaplasma phagocytophilum* IgG antibodies were found in 9.6% of participants, at the titers 1:64 and 1:128 in 39 and one serum sample, respectively. Antibodies against *Bartonella henselae* were detected in 193 of 416 tested serum samples (46.4%), at the titers 1:64, 1:128, and 1:256 in 182, 10, and one serum sample, respectively. The presence of IgG antibodies against *Yersinia enterocolitica*/*Y. pseudotuberculosis* was detected in 43.4% of the tested samples. In research conducted on a group of participants, the seroprevalence rates of *M. pneumoniae* IgM and IgG were 8.9% and 63.6%, respectively. In research conducted on the presented project participants, only *C. pneumoniae* IgG antibodies were detected, with a seroprevalence rate of 47.4% (Table 3).

## 4. Discussion

Using ELISA, this study showed the seroprevalence of *B. burgdorferi* s.l. in the population related to the rural environmental to be 33.8%, whereas 58.7% of results were confirmed using immunoblot. Currently, the standard for diagnosing LB is two-tiered serology, ELISA and immunoblot [14]. To detect specific anti-*Borrelia burgdorferi* antibodies, we used tests containing recombinant antigens, which are characterized by high sensitivity and specificity. According to Moniuszko-Malinowska et al. [11], recombinant immunoblot assays have significantly higher sensitivity than conventional immunoblot, combined with equally high specificity. Even with two-step diagnostics, certain limitations may occur. The sensitivity of the tests depends on the stage of the disease, therapy used, and the ability of bacteria to evade the immune system. Moreover, false positive results may be associated with, among other things, cross-reactions with other pathogens or autoimmune disorders [15]. Of the more than 41% of concurrently positive ELISA and negative immunoblot results, the vast majority were only slightly above the cut-off value in ELISA. Additionally, the western blot test does not eliminate all false positive results but significantly reduces their occurrence. In Europe, LB seroprevalence estimates vary by country and region from 0 to 70%, with the highest values for the general population found in eastern regions [16]. Such differences in the occurrence of specific antibodies against *B. burgdorferi* s.l. noted in various reports depend on many factors related to the research methodology (including the use of different tests and study groups), as well as environmental conditions (including the prevalence of the pathogen in the environment).

Seroprevalence of *Toxoplasma gondii* also varies between countries and regions depending on age, habits, diet, and environmental factors [17,18]. In Europe, mandatory screening is carried out in Austria, France, Belgium, Slovakia, Slovenia, Romania, Croatia, Italy, Serbia, and Poland but only for pregnant women. For other target groups (e.g., HIV-positive patients, hospital patients, organ donors, and transplant recipients), active surveillance is also carried out depending on the country [19,20]. However, in Poland, data on the *T. gondii* prevalence in the occupational groups exposed to contact with the parasite (e.g., farmers, veterinarians) are limited and mostly presented on the basis of our own research [21,22]. In our study, over 50% of serum samples were positive in the IgG class. Similar IgG results were found in the past in forestry workers (59%) and farmers (57%) from the Lublin region of eastern Poland [23], and veterinarians (45.5%) from 12 provinces of Poland [24]. However, this seroprevalence did not exceed the average values recorded in Poland. Due to direct contact with infected animals or raw meat, veterinarians and animal breeders may be perceived as groups at risk of contact with the parasite [25]. Despite the relatively high percentage of positive IgG results in this study, specific IgM was detected in only 1.4% people, which is closer to the value of 5% obtained by Nowakowska et al. in 2006 [26].

In Europe, the number of diagnosed clinical cases of human granulocytic anaplasmosis is much lower than in the USA, but, at the same time, the seroprevalence of HGA antibodies in Europe is increasing up to 31.0% (among individuals suspected of tick-borne infection) [27]. The relatively high seroprevalence from the current study may result from participation in the study group of people living or working in an endemic region for tick-borne diseases (eastern Poland). Cross-reactivity with other pathogens therefore cannot be excluded. In Poland, single cases of anaplasmosis are reported every year [28]. However, a recent study (2011) by Moniuszko-Malinowska et al. [29] shows that the number of diagnosed cases of HGA may be underestimated. Of the 1375 patients from north-eastern Poland with suspected tick-borne disease, 120 (8.7%) were diagnosed with anaplasmosis. 

Serological investigations for *Yersinia enterocolitica*/*Y. pseudotuberculosis* in the healthy population in Europe are rare, with most of the results originating from the 2000s and earlier. These few publications have reported high seropositive results (19–43%) in healthy humans (including blood donors) in Finland, Germany, Austria, and Ireland [30,31,32], which is in accordance with the value obtained in the current study. The EFSA reported a yersiniosis notification rate of 2.2 per 100,000 population in 2022 (7919 confirmed cases) [7]. High seroprevalence suggests that the number of infections may be higher, and the disease may be underdiagnosed due to diagnostic difficulties or the asymptomatic or mild nature of the infection. Asymptomatic *Yersinia* infections are particularly dangerous due to the risk of post-transfusion sepsis (with even fatal outcomes) as a result of blood transfusion derived from a seemingly healthy donor [33].

In the current study, antibodies against *Bartonella henselae* were detected in 193 of 416 tested serum samples (46.4%), which is consistent with the results from other European countries. A similar seroprevalence rate for *B. henselae* was noted in Croatia (41.3 and 57.4% in children and blood donors, respectively) [34], Spain (37.1 and 53.6% in veterinary personnel and sanitary workers, respectively) [35,36], Slovakia (23.5% in the general population) [37], and Germany (35.5 and 45.3% in office workers and forestry workers, respectively) [38]. In the current study, most participants declared they were farmers or lived in rural areas, where they were likely exposed to contact with animals (e.g., cats) and, thus, with their external parasites, mainly fleas, responsible for transmission of bacteria [39]. All patients were seronegative for *B. quintana*, which is mainly transmitted by body lice [5].

*Mycoplasma pneumoniae* is a common causative agent of tracheobronchitis and atypical pneumonia, mainly in children and adolescents in the autumn–winter seasons. In Poland, *M. pneumoniae* is responsible for 30–40% of all cases of bacterial respiratory infections [40]. In 2019, the IgG and IgM seroconversion rates against *M. pneumoniae* in South Korea were 16.7% and 33.3%, respectively [41]. The latest study, conducted in 2018–2019, which included a group of children aged 0–12 years, showed the prevalence of antibodies against *M. pneumoniae* at 14.6%. This may indicate that *M. pneumoniae* infection may also be common at younger ages [42].

The prevalence of antibodies to three chlamydial species, *Chlamydia pneumoniae*, *C. psittaci*, and *C. trachomatis,* has rarely been studied, especially when it concerns potentially healthy people. In the general population in China (1996), IgG antibodies against *C. pneumoniae*, *C. trachomatis*, and *C. psittaci* were present in 61.5%, 9.3%, and 3.5%, respectively, and increased with age [43]. In Italy (2002), the seroprevalence of *C. pneumoniae* was tested in stable asthmatic patients. Among them, IgG antibodies were detected in 30.4% of the patients and 30.8% of people from the control group, which did not confirm a relationship between the level of *C. pneumoniae* antibodies and stable asthma [44]. In Poland (2005), research conducted among children indicated that *C. pneumoniae* may play a role in the aetiology of respiratory tract infections. IgM antibodies were detected in 13.0% of children with respiratory tract infections, while IgG antibodies were found in the sera of 11 children under 12 months old [45]. Other studies indicate a positive correlation between cardiovascular disease and the presence of *C. pneumoniae* antibodies [41,46]. *Chlamydia pneumoniae* infection is an important factor of morbidity and mortality among older adults with anti-*C. pneumoniae* antibodies confirmed as high [47].

Positive results for specific IgM antibodies detected for causative agents of Lyme borreliosis, mycoplasmosis, and toxoplasmosis can be presumptive evidence of recent infection. In the case of Lyme borreliosis, the presence of so-called persistent IgM antibodies cannot be excluded. Positive results for specific IgM antibodies for more than a month without seroconversion to IgG may be a false positive result. Specific IgG antibodies should appear in patients infected with *B. burgdorferi* s.l. spirochetes a few weeks after infection [11]. Positive results for specific IgG antibodies alone may indicate that the patient has no current infection. Re-infection after primary infection with infectious agents cannot be ruled out [48,49].

A major limitation in the choice of research method is the limited access to tests approved for in vitro diagnostics (IVD). *M. pneumoniae* and *C. pneumoniae* can be cultured from respiratory secretions, but diagnosis of atypical pneumonias is most often serological [50]. For both tests used, the manufacturers declared high sensitivity and specificity. The IFA tests used in our research are characterized by sensitivity and specificity above 96%, although cross-reactivity between species for *Bartonella* may occur. The indirect immunofluorescence method is recommended for anaplasmosis and bartonellosis diagnostics by the Polish Society of Epidemiologists and Infectious Disease Physicians [13,51]. Cell line or microbiological cultures are performed in few reference laboratories, and this method is time-consuming. Molecular analyses are characterized by high sensitivity and specificity but also have limitations, such as the type of clinical specimen and bacterial loads in body fluids. Serological methods are recommended in the diagnosis of toxoplasmosis [12]. ELFA and WB are characterized by high specificity and specificity. Previous studies have confirmed the effectiveness of the tests in detecting antibodies against *T. gondii* in pregnant women [52]. The basic diagnostic method for gastrointestinal infections is the isolation of *Y. enterocolitica* or *Y. pseudotuberculosis* from clinical material. A stool culture is the best way to confirm the diagnosis. Molecular tests are performed, and, in the diagnosis of extraintestinal infections like reactive arthritis, antibody titers are also tested. The single IgG-specific ELISA for *Yersinia* exhibits high sensitivity but low specificity. Due to the possibility of cross-reactions with other bacteria, it is necessary to perform additionally testing, like combining testing IgG with testing IgM and IgA. The IgM and IgA ELISA tests demonstrate higher specificity, 100 and 89%, respectively. The best choice will be Western blot, which has high parameters (95% sensitivity and specificity) and is useful in the diagnosis of enteric infections and reactive arthritis [53]. It should be emphasized that, in all cases, clinical diagnosis should be based on the complete clinical history and laboratory findings.

A limitation of the presented study that could have influenced the results was that the samples were also collected in 2020 when the COVID-19 pandemic began in Poland. The changes in people’s behaviour resulting from actions taken by the authorities and individuals to limit the spread of the SARS-CoV-2 virus may have influenced some of our results. We have observed lower seropositive results for *M. pneumoniae* and *C. pneumoniae* in 2020 compared to 2019 (Table 4). These pathogens are responsible for community-acquired respiratory infections. During the pandemic, campaigns were conducted to raise awareness among residents about preventing the spread of respiratory diseases. There was an increased focus on hygiene (including the use of disinfectants) and the use of personal protective equipment, such as masks and gloves, etc. Moreover, as the virus spread, person-to-person contact decreased [54]. These activities could have resulted in a lower rate of occurrence of antibodies to *M. pneumoniae* and *C. pneumoniae* in the study population in 2020. Moreover, in 2020, there were reports of co-infection of the Sars-cov-2 virus with *C. pneumoniae* or *M. pneumoniae*. The double infection may influence the course of the disease [55]. In the current study, the seroprevalence rate of *B. burgdorferi* s.l. and *B. henselae* also decreased between 2019 and 2020, from 23.3 to 14% and 56.6 to 36.3%, respectively (Table 4). This difference may not be strictly related to the pandemic alone but also due to the larger number of foresters tested in 2019 (42) compared to 2020 (12), which is justified by the occupational exposure to tick bites associated with this group [13]. Additionally, in 2020, the vast majority of samples were collected in the second quarter of the year (207 out of 216), while, in 2019, most samples were collected in the third quarter (202 out of 257). Analysing the number of Lyme borreliosis cases reported annually in Poland [28], the lowest number of reports were recorded in the first and second quarters.

## 5. Conclusions

Among the infectious diseases studied, a higher seroprevalence in the human population was found in the case of factors accompanying respiratory diseases than in the case of tick-borne diseases. Our results also suggest a high prevalence of *T. gondii* antibodies (51.7% for IgG) among participants occupationally exposed. The rural environment is the main source of diseases, including parasitological, respiratory, and tick-borne diseases. The results regarding the prevalence of selected infectious diseases may draw the attention of public health authorities to the need for implementing educational programmes to monitor and reduce the transmission of infectious diseases.

## Figures and Tables

**Table 1:** Seroprevalence of *Borrelia burgdorferi* s l. in the human population (eastern Poland).

Participants		ELISA							Western blot					
		IgM		IgG		Total			IgM		IgG		Total	
Age	N	Pos	% (95% CI)	Pos	% (95% CI)	Pos	% (95% CI)	N	Pos	% (95% CI)	Pos	% (95% CI)	Pos	% (95% CI)
≤30	45	12	26.7 (14.6–41.9)	2	4.4 (0.5–15.1)	13	28.9 (16.4–44.3)	45	5	11.1 (3.7–24.0)	2	4.4 (0.5–15.1)	6	13.3 (5.0–26.8)
31–40	73	14	19.2 (10.9–30.1)	10	13.7 (6.8–23.7)	21	28.8 (18.8–40.5)	72	8	11.1 (4.9–20.7)	6	8.3 (3.1–17.3)	12	16.7 (8.9–27.3)
41–50	112	21	18.7 (12.0–27.2)	18	16.1 (9.8–24.2)	29	25.9 (18.1–35.0)	112	18	16.1 (9.8–24.2)	14	12.5 (7.0–20.1)	24	21.4 (14.2–30.3)
51–60	111	27	24.3 (16.7–33.4)	19	17.1 (10.6–25.4)	37	33.3 (24.7–42.9)	106	11	10.4 (5.3–17.8)	10	9.4 (4.6–16.7)	18	17.0 (10.4–25.5)
61–70	81	16	23.5 (14.7–34.2)	30	37.0 (26.6–48.5)	38	46.91 (35.7–58.3)	77	14	18.2 (10.3–28.6)	22	28.6 (18.8–40.0)	29	37.7 (26.9–49.4)
>71	42	7	16.7 (7.0–31.4)	17	40.5 (25.6–56.7)	19	45.2 (29.8–61.3)	38	5	13.2 (4.4–28.1)	7	18.4 (7.7–34.3)	10	26.3 (13.4–43.1)
Total	464	97	20.9 (17.3–24.9)	96	20.7 (17.1–24.7)	157	33.8 (29.5–38.3)	450	58	12.9 (9.9–16.3)	61	13.6 (10.5–17.1)	99	22.0 (18.3–26.1)

N—number of tested samples; Pos—number of positive results; CI—confidence interval.

**Table 2:** Seroprevalence of Toxoplasma gondii in the human population (eastern Poland).

Participants		IgM		IgG		IgG avidity						
		Pos	% (95% CI)	Pos	% (95% CI)	N	H		M		L	
Age	N						Pos	% (95% CI)	Pos	% (95% CI)	Pos	% (95% CI)
≤30	45	1	2.2 (0.1–11.78)	5	11.1 (3.7–24.1)	4	4	100 (39.8 ^a^–100)	0	0.0 (0.0–60.2 ^a^)	0	0.0 (0.0–60.2 ^a^)
31–40	69	1	1.4 (0.0–7.8)	26	37.7 (26.3–50.2)	25	25	100 (86.3 ^a^–100)	0	0.0 (0.0–13.7 ^a^)	0	0.0 (0–13.7 ^a^)
41–50	108	2	1.8 (0.2–6.5)	49	45.4 (35.8–55.2)	44	42	95.4 (84.5–99.4)	1	2.3 (0.1–12.0)	1	2.3 (0.1–12.0)
51–60	104	1	1.0 (0.0–5.2)	68	65.4 (55.4–74.4)	56	53	94.6 (85.1–98.9)	3	5.4 (1.1–14.9)	0	0.0 (0.0–6.4 ^a^)
61–70	63	0	0.0 (0.0–5.7 ^a^)	45	71.4 (58.6–82.1)	36	34	94.4 (81.3–99.3)	2	5.6 (0.7–18.7)	0	0.0 (0.0–9.7 ^a^)
>71	33	1	3.0 (0.1–15.78)	25	75.8 (57.7–88.9)	22	20	90.9 (70.8–98.9)	2	9.1 (1.1–29.2)	0	0.0 (0.0–15.4 ^a^)
Total	422	6	1.4 (0.5–3.1)	218	51.7 (46.8–56.5)	187	178	95.2 (91.1–97.8)	8	4.3 (1.9–8.2)	1	0.5 (0.0–2.9)

N—number of tested samples; Pos—number of positive results; H—high avidity antibodies; M—medium avidity antibodies; L—low avidity antibodies; CI—confidence interval; ^a^—One-sided 97.5% confidence interval.

**Table 3:** Seroprevalence of *Anaplasma phagocytophilum*, *Bartonella* spp., *Mycoplasma pneumoniae,* and *Chlamydia pneumoniae* in the human population (eastern Poland).

Participants	*Bartonella henselae* (IgG)		*Anaplasma phagocytophilum* (IgG)		*Yersinia enterocolitica*/*Y. pseudotuberculosis* (IgG)		*Mycoplasma pneumoniae* (IgM)		*Mycoplasma pneumoniae* (IgG)		*Chlamydia pneumoniae* (IgG)	
	P/N	% (95% CI)	P/N	% (95% CI)	P/N	% (95% CI)	P/N	% (95% CI)	P/N	% (95% CI)	P/N	% (95% CI)
≤30	22/42	52.4 (36.4–68.0)	4/40	10.0 (2.8–23.7)	13/42	30.9 (17.6–47.1)	8/42	19.0 (8.6–34.1)	28/42	66.7 (50.4–80.4)	12/42	28.6 (15.7–44.6)
31–40	42/67	62.7 (50.0–74.2)	8/67	11.9 (5.3–22.2)	22/68	32.3 (21.5–44.8)	8/67	11.9 (5.3–22.2)	43/67	64.2 (51.5–75.5)	21/68	30.9 (20.2–43.3)
41–50	43/105	40.9 (31.4–51.0)	9/105	8.6 (4.0–15.6)	45/106	42.4 (32.9–52.4)	10/104	9.6 (4.7–17.0)	65/104	62.5 (52.5–71.8)	47/105	44.8 (35.0–54.8)
51–60	44/99	44.4 (34.4–54.8)	6/99	6.1 (2.3–12.7)	48/98	49.0 (38.7–59.3)	9/99	9.1 (4.2–16.6)	69/99	69.7 (59.6–78.5)	53/97	54.6 (44.2–64.8)
61–70	30/68	44.1 (32.1–56.7)	8/69	11.6 (5.1–21.6)	35/68	51.5 (39.0–63.8)	1/68	1.5 (0.0–7.9)	40/68	58.8 (46.2–70.6)	37/70	52.9 (40.5–64.9)
>71	12/35	34.3 (19.1–52.2)	5/36	13.9 (4.7–29.5)	18/35	51.4 (34.0–68.6)	1/35	2.9 (0.1–14.9)	19/35	54.3 (36.6–71.2)	28/36	77.8 (60.8–89.9)
Total	193/416	46.4 (41.5–51.3)	40/416	9.6 (7.0–12.9)	181/417	43.4 (38.6–48.3)	37/415	8.9 (6.4–12.1)	264/415	63.6 (58.8–68.2)	198/418	47.4 (42.5–52.3)

N—number of tested samples; P—number of positive results; CI—confidence interval.

**Table 4:** Seroprevalence of Borrelia burgdorferi sensu lato, Anaplasma phagocytophilum, Bartonella henselae, Mycoplasma pneumoniae, Chlamydia pneumoniae, and Toxoplasma gondii depends on the year of sample collection.

	*Borrelia burgdorferi* Sensu Lato * (IgM/IgG)	*Bartonella henselae* *** (IgG)	*Anaplasma phagocytophilum* (IgG)	*Yersinia enterocolitica*/*Y. pseudotuberculosis* (IgG)	*Mycoplasma pneumoniae* *** (IgM/IgG)	*Chlamydia pneumoniae* ** (IgG)	*Toxoplasma gondii* (IgM/IgG)
Year of Sampling	Pos/N % (95% CI)	Pos/N % (95% CI)	Pos/N % (95% CI)	Pos/N % (95% CI)	Pos/N % (95% CI)	Pos/N % (95% CI)	Pos/N % (95% CI)
2019	53/227 23.3 (18.0–29.4)	116/204 56.9 (49.7–63.8)	20/204 9.8 (6.1–17.7)	94/205 45.9 (38.9–52.9)	152/203 74.9 (68.3–80.7)	115/206 55.8 (48.8–62.7)	109/209 52.2 (45.2–59.1)
2020	30/215 14 (9.6–19.3)	77/212 36.3 (29.8–43.2)	20/212 9.4 (5.9–14.2)	87/212 41.0 (34.4–48.0)	118/212 55.7 (48.7–62.5)	83/212 39.2 (32.5–46.1)	109/213 51.2 (44.3–58.1)

Association between prevalence of seropositive reactions was analysed by chi-squared test, depending on year of sampling: * result significant at *p* < 0.05, ** result significant at *p* < 0.001, *** result significant at *p* < 0.0001.

## Data Availability

All relevant data are provided in the manuscript. Raw data can be made available upon reasonable request.
